# Knockdown of DNA methyltransferase 1 reduces DNA methylation and alters expression patterns of cardiac genes in embryonic cardiomyocytes

**DOI:** 10.1002/2211-5463.13252

**Published:** 2021-07-24

**Authors:** Xiefan Fang, Ryan Poulsen, Lu Zhao, Jingjing Wang, Scott A. Rivkees, Christopher C. Wendler

**Affiliations:** ^1^ Department of Pediatrics Child Health Research Institute College of Medicine University of Florida Gainesville FL USA; ^2^ Charles River Laboratories, Inc. Reno NV USA

**Keywords:** DNA methylation, DNMT1, embryonic cardiomyocyte, gene expression

## Abstract

We previously found that DNA methyltransferase 3a (DNMT3a) plays an important role in regulating embryonic cardiomyocyte gene expression, morphology, and function. In this study, we investigated the role of the most abundant DNMT in mammalian cells, DNMT1, in these processes. It is known that DNMT1 is essential for embryonic development, during which it is involved in regulating cardiomyocyte DNA methylation and gene expression. We used siRNA to knock down DNMT1 expression in primary cultures of mouse embryonic cardiomyocytes. Immunofluorescence staining and multielectrode array were, respectively, utilized to evaluate cardiomyocyte growth and electrophysiology. RNA sequencing (RNA‐Seq) and multiplex bisulfite sequencing were, respectively, performed to examine gene expression and promoter methylation. At 72 h post‐transfection, reduction of DNMT1 expression decreased the number and increased the size of embryonic cardiomyocytes. Beat frequency and the amplitude of field action potentials were decreased by *DNMT1* siRNA. RNA‐Seq analysis identified 801 up‐regulated genes and 494 down‐regulated genes in the DNMT1 knockdown cells when compared to controls. Pathway analysis of the differentially expressed genes revealed pathways that were associated with cell death and survival, cell morphology, cardiac function, and cardiac disease. Alternative splicing analysis identified 929 differentially expressed exons, including 583 up‐regulated exons and 308 down‐regulated exons. Moreover, decreased methylation levels were found in the promoters of cardiac genes *Myh6*, *Myh7*, *Myh7b*, *Tnnc1*, *Tnni3*, *Tnnt2*, *Nppa*, *Nppb*, *mef2c*, *mef2d*, *Camta2*, *Cdkn1A*, and *Cdkn1C*. Of these 13 genes, 6 (*Myh6*, *Tnnc1*, *Tnni3*, *Tnnt2*, *Nppa*, *Nppb*) and 1 (*Cdkn1C*) had increased or decreased gene expression, respectively. Altogether, these data show that DNMT1 is important in embryonic cardiomyocytes by regulating DNA methylation, gene expression, gene splicing, and cell function.

## Graphical abstract

Knockdown of DNMT1 expression in mouse embryonic cardiomyocytes decreased promoter DNA methylation levels, altered activities of transcription factors, changed gene expression and splicing, and decreased cell viability, beating frequency, and amplitude of field action potential.

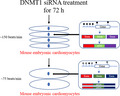

**Abbreviations**
DAVID: Database for Annotation, Visualization and Integrated Discovery
DE: differential expression
DEU: differential exon usage
DNMT: DNA methyltransferase
FDR: false discovery rate
IPA: Ingenuity Pathway Analysis
RNA: RNA sequencing
siRNA: small interfering RNA

Cardiomyocyte development is an orchestrated process with characteristic gene expression profiles at different stages [1]. Cardiomyocytes also respond to postnatal and pathological conditions with signature gene expression patterns [2]. Expression of these genes is tightly controlled by mechanisms including transcription factors and chromatin modifications [3, 4]. DNA methylation is also a major modulator of cardiac gene expression [5, 6]. After differentiation, cardiomyocytes acquire signature DNA methylation patterns that are distinct from other cell types [7]. DNA methylation changes are dynamic during cardiomyocyte development, maturation, and disease [5], which correlate with gene expression changes.

DNA methyltransferases (DNMTs) and TET methylcytosine dioxygenases (TETs) are responsible for the establishment and maintenance of DNA methylation patterns [8, 9]. Among the three DNMT isoforms, DNMT1 is the most abundant in gene expression. In embryonic day (E) 13.5 mouse embryonic heart, the expression of *Dnmt1* mRNA is 14 times higher than that of *Dnmt3a* and 160 times higher than that of *Dnmt3b* [10]. The major role of DNMT1 is to maintain methylation patterns during cell division [11]. DNMT3a and 3b are primarily *de novo* DNMTs that play an important role in establishing DNA methylation patterns during early embryogenesis [11]. DNMTs are essential for development, and deletion of DNMT1 or DNMT3b is embryonic lethal [12, 13]. In general, methylation of normally unmethylated CpG sites that are located in the 5′ promoter region is associated with transcriptional inactivation of many genes [14]. Gene body methylation also contributes to gene regulation and alternative splice [15, 16, 17]. Importantly, altered DNA methylation patterns can be stably inherited during DNA replication and mediate persistent toxicological consequences in subsequent cellular and animal generations [18, 19, 20, 21, 22, 23].

More and more environmental factors or chemicals have been identified to be capable of modifying the DNA methylome via inducing DNMT1 mutation or altering DNMT1 expression [24, 25, 26, 27]. Meanwhile, a growing body of evidence demonstrates a link between aberrant DNA methylation and cardiovascular diseases (CVDs) in humans [28, 29, 30, 31, 32, 33, 34]. However, by far, the significance of DNMT1 in regulating gene expression in cardiomyocytes remains largely unknown. Thus, it is important to understand the role of DNA methylation in the control of cardiac gene expression and function. Recently, it is reported that pharmacological inhibition of DNA methylation attenuates pressure overload‐induced cardiac hypertrophy in rats [35]. In addition, it has been demonstrated that myocardial tissue‐specific DNMT1 knockout in rats protects against pathological injury induced by Adriamycin [36]. These studies suggest that decreased DNMT1 expression and the subsequent DNA hypomethylation may play a protective role against pathological cardiac changes.

Our previous studies find that DNMT3a plays an important role in regulating gene expression, cardiomyocyte function, and morphology [10]. Knockdown of DNMT3a disrupts sarcomere assembly and decreases beating frequency, contractile movement, amplitude of field action potential, and cytosolic calcium signaling of embryonic cardiomyocytes. The DNMT3a knockdown cells have abnormal gene expression and DNA methylation patterns. However, the influences of DNMT1, the most abundant DNMT, on gene expression and DNA methylation in embryonic cardiomyocytes remained unexplored.

To investigate the role of DNMT1, we used siRNAs to knock down DNMT1 expression in embryonic mouse cardiomyocytes, which were examined for viability, contractility, electrophysiology, whole‐genome transcription levels, and target gene promoter methylation patterns. We now demonstrate that disruption of DNMT1 activity directly influences cardiomyocyte promoter methylation, gene expression and splicing, cell survival, and contractility.

## Materials and methods

### Materials

Fibronectin and monoclonal anti‐α‐actinin (sarcomeric) antibody (#A7811) were purchased from Sigma‐Aldrich (St. Louis, MO, USA). Dulbecco's modified Eagles media (DMEM), inactivated fetal bovine serum, antibiotic‐antimycotic solution, goat anti‐mouse IgG Alexa 488 secondary antibody, 4', 6‐diamidino‐2‐phenylindole dihydrochloride (DAPI), Lipofectamine RNAiMAX, RNAqueous^®^‐Micro Total RNA Isolation Kit, and Power SYBR^®^Green PCR Master Mix were ordered from Thermo Fisher Scientific (Waltham, MA, USA). Accutase and Accumax were purchased from Innovative Cell Technologies (San Diego, CA, USA). FlexiTube GeneSolution (with 4 siRNAs) DNMT1 siRNA (#GS13433) and AllStars negative control siRNA (#1027281) were obtained from Qiagen (Valencia, CA, USA). Quick‐gDNA™ MicroPrep Kit, ZymoTaq™ PreMix (Hot start DNA Taq polymerase), and ZR‐96 DNA Clean & Concentrator™‐5 Kit were purchased from Zymo Research (Irvine, CA, USA). NEBNext^®^ mRNA Library Prep Master Mix Set for Illumina and the NEBNext Multiplex Oligos for Illumina were ordered from New England BioLabs (Ipswich, MA, USA). iScript cDNA Synthesis Kit was ordered from Bio‐Rad (Hercules, CA, USA). Nextera XT DNA Library Preparation Kit, Nextera XT Index Kit, and 20% PhiX control libraries were obtained from Illumina (San Diego, CA, USA).

### Isolation, culture, and treatment of primary embryonic cardiomyocytes

All animal experiments were approved by the Institutional Animal Care and Use Committee (IACUC) of the University of Florida. CD‐1 mice, at least 8 weeks old, were purchased from Charles River Laboratories (Wilmington, MA, USA). Mice were housed in same‐sex cages and maintained in a temperature/humidity‐controlled room with 12‐h light/dark cycle. Mice had access to food and water ad libitum. Timed mating was performed, and the day a vaginal plug was observed was designated as embryonic day 0.5 (E0.5). Primary cardiomyocytes were isolated from mouse E13.5 embryonic ventricles, as described [37]. Cardiomyocytes were cultured in DMEM supplemented with 10% inactivated fetal bovine serum, 2 mm l‐glutamine, and antibiotic‐antimycotic solution.

Cardiomyocytes were seeded at a density of 6.0 × 10^5^ cells per well in 12‐well culture plates or at 6.0 × 10^6^ per 12.5 mm^2^ flask, as described [10]. Cells were cultured for 48 h at 37 °C to reach 70–80% confluency. DNMT1 (12 nm or 24 nm) or negative control siRNA was transfected into the cells using Lipofectamine RNAiMAX.

### Immunofluorescence staining and cell imaging

Cardiomyocytes were stained with α‐actinin antibody and DAPI as described [37]. A Cytation 5 Cell Imaging Multi‐Mode Reader (BioTek, Winooski, VT, USA) was used at a montage mode to scan the cardiomyocytes. The total cell number (DAPI‐positive cells), number of cardiomyocytes (α‐actinin and DAPI‐positive cells), percent cardiomyocyte (α‐actinin‐positive cells/DAPI‐positive cells), and averaged size of cardiomyocytes (α‐actinin‐positive area/number of cardiomyocytes) were calculated with the Gen5™ software (BioTek) and an in‐house R script.

### Field potential recordings

Field potential was recorded as described [10]. Primary cardiomyocytes were seeded on the 60PedotMEA200/30iR‐Au multielectrode arrays (Multichannel Systems, Reutlingen, Germany) coated with 10 µg·mL^−1^ fibronectin, allowed to grow to 70–80% confluency, and treated with DNMT1 siRNA or negative siRNA. During 3–7 days post‐transfection, electrical activity was measured daily using a MEA2100 amplifier (Multichannel Systems) at a sampling rate at 20 KHz, with temperature kept at 37 °C. Data were recorded and analyzed with the mcrack software (Multichannel Systems).

### Sorting of cardiomyocytes and isolation of RNA and DNA

Cardiomyocytes were treated with 12 nm DNMT1 or negative siRNAs, released with Accutase, and dissociated with Accumax. Cardiomyocytes were sorted as described [10]. Briefly, dissociated cells were stained with anti‐VCAM1 antibody conjugated with allophycocyanin (APC; BioLegend, San Diego, CA, USA), followed by magnetic sorting using anti‐APC microbeads and magnetic assisted cell sorting (MACS) columns (Miltenyi Biotec, Bergisch Gladbach, Germany).

Total RNA was isolated from the sorted cardiomyocytes with the RNAqueous^®^‐Micro Total RNA Isolation Kit. Genomic DNA was extracted with the Quick‐gDNA™ MicroPrep Kit, and DNA concentrations were quantitated with NanoDrop 2000 (Thermo Fisher Scientific).

### Illumina transcriptomic RNA sequencing (RNA‐Seq)

RNA‐Seq was performed as described [10]. mRNA was isolated from total RNA using NEXTflex™ Poly(A) Beads (Bioo Scientific, Austin, TX, USA). Sequencing libraries were prepared with the NEBNext^®^ mRNA Library Prep Master Mix Set for Illumina and the NEBNext Multiplex Oligos for Illumina. Illumina‐adapted libraries, including cardiomyocyte samples treated with negative or DNMT1 siRNA (*n* = 3/treatment), were pooled at equal molar ratio and sequenced with one High Output 1 × 75 cycles run on a NextSeq500 sequencer (Illumina, San Diego, CA, USA). All RNA‐Seq data were uploaded to the Gene Expression Omnibus (GEO), and the accession number is GSE81446.

### RNA‐Seq data analysis for differential gene expression

RNA‐Seq data analysis was performed as described [10]. Briefly, the fastq files generated from RNA‐Seq were uploaded to the UF Research Computing Galaxy instance developed by the University of Florida. The data were cleaned with the FastQC program and mapped to the mouse genome (mm10) with the Tophat2 tool. Counting of RNA‐seq reads was performed with HTSeq [38]. Differential expression (DE) of genes between treatments was analyzed using two methods: r packages edger [39] and deseq2 [40], with Ensembl Mus_GRCm38.79.gtf as the reference annotation. Genes with false discovery rate (FDR) < 0.05 and absolute fold change > 1.5 were considered as significant. Unique DE genes were identified by combining the results generated from the two analytical methods. Functional ontology was conducted with the unique DE genes using the Database for Annotation, Visualization, and Integrated Discovery (DAVID) [41] and Ingenuity Pathway Analysis (IPA; Qiagen) [42]. The significance criterion for pathway analysis was *P* < 0.05.

### RNA‐Seq data analysis for differential exon usage (DEU)

As described [10], DEU analysis was performed with R package DEXSeq to identify changes in the relative usage of each exon following siRNA treatments [43]. Exons with FDR < 0.05 and absolute fold change > 1.5 were considered as significant. Functional ontology analysis on DEU genes was conducted with DAVID.

DE and DEU genes were compared, and the genes affected in both expression and alternative splicing by siRNA treatments were identified.

### Quantitative real‐time PCR (qPCR) analysis

Total RNA was reverse transcribed to cDNA libraries by using iScript cDNA Synthesis Kit. Power SYBR^®^Green PCR Master Mix was used to perform qPCR analysis in a GeneAmp 7300 Real‐Time PCR System (18). Target genes were amplified using published qPCR primer pairs (18). β‐actin primers were used as an internal control.

### Sodium bisulfite PCR amplification of target gene regions

Genomic DNA was treated with sodium metabisulfite for bisulfite conversion, as described [44]. Using published bisulfite specific primers [10], 15 target genes (two promoter regions per gene) were PCR‐amplified from the bisulfite‐converted DNA with ZymoTaq™ PreMix (Hot start DNA Taq polymerase). These genes are related to cardiomyocyte morphology and proliferation and included *Myh6*, *Myh7*, *Myh7b*, *Tnnc1*, *Tnni3*, *Tnnt2*, *Nppa*, *Nppb*, *mef2c*, *mef2d*, *Camta1, Camta2*, *Cdkn1A*, *Cdkn1B*, and *Cdkn1C*. PCR products were cleaned with the ZR‐96 DNA Clean & Concentrator™‐5 Kit.

### Illumina DNA library preparation and sequencing

PCR products from the same genomic DNA were pooled at equal concentration ratio and made into an indexed sequencing library with the Nextera XT DNA Library Preparation Kit and the Nextera XT Index Kit, as described [10]. Illumina‐adapted libraries, including cardiomyocyte samples treated with negative or 12 nm DNMT1 siRNA (*n* = 3/treatment), were pooled at equal molar ratio, spiked with 20% PhiX control libraries, and sequenced with one 1 × 150 cycles run (v3) on a MiSeq sequencer (Illumina). All bisulfite‐Seq data were uploaded to GEO, and the accession number is GSE81464.

### Multiplex bisulfite sequencing data analysis

The fastq files generated from a MiSeq sequencer were uploaded to the UF Research Computing Galaxy instance, as described [10]. The data were cleaned with the FastQC program. A reference genome with the amplicon sequences was built and bisulfite‐converted *in silico* with Bismark Bisulfite Mapper [45]. The high‐quality sequence reads were aligned to the reference genome. Cytosine methylation (CpG) counting was performed with the Bismark methylation extractor. Differential methylation was analyzed with the Methylkit package [46]. CpG sites with FDR <0.05 and absolute percent methylation difference >5% were considered as significant.

### Statistical analysis

Results were analyzed using graphpad prism 6.0 (GraphPad Software, La Jolla, CA, USA). All experiments were performed in triplicate. Data are presented as mean ± SEM. Statistical differences between treatment groups were determined using Student’s *t*‐test or one‐way ANOVA followed by Neuman–Keuls *post hoc* test. A *P*‐value < 0.05 was considered statistically significant.

## Results

### Down‐regulation of DNMT1 expression decreases the number and increases size of embryonic cardiomyocytes

To investigate the role of DNMT1 in regulating cardiomyocyte physiology, we reduced DNMT1 expression by using pooled siRNAs in cultured E13.5 cardiomyocytes. Our previous studies find that during 2–5 days post‐siRNA transfection, 12 nm DNMT1 siRNA decreases DNMT1 mRNA and protein expression by > 70%, with a transfection efficiency near 100% [10]. In this study, we also confirmed about 75% and 92% knockdown of DNMT1 mRNA at 72 h post‐transfection by 12 nm and 24 nm siRNA, respectively (data not shown).

**Fig. 1:**
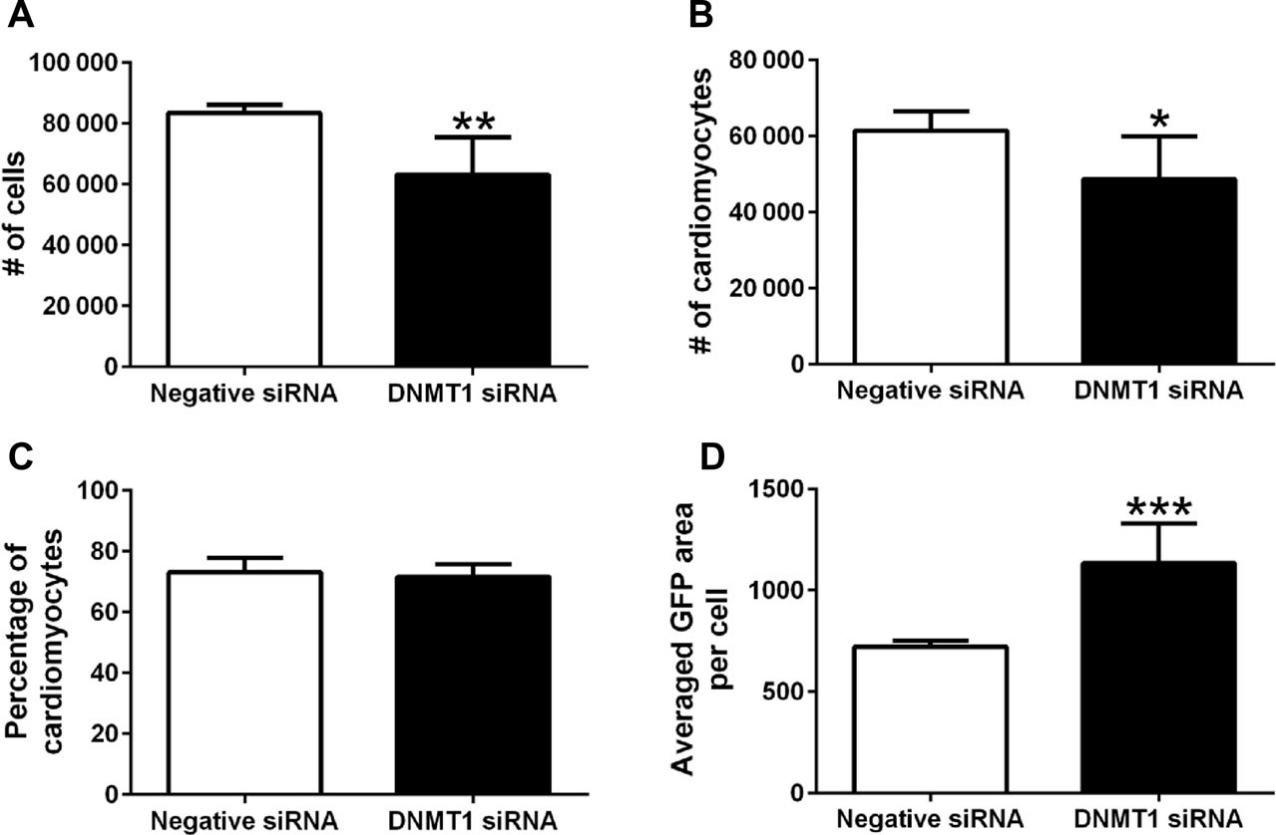
Knockdown of DNMT1 expression reduces the number and increases the size of cardiomyocytes. At 72 h post‐siRNA transfection, the primary cardiomyocytes transfected with 12 nm DNMT1 siRNA or negative siRNA were stained with α‐actinin (green) and DAPI (blue) and imaged by a Cytation 5 Cell Imaging Multi‐Mode Reader (images not shown). Within the interrogated regions, the Gen5™ software (BioTek) and an in‐house R script were used to calculate the number of total cells (DAPI‐positive cells; A), number of cardiomyocytes (DAPI and α‐actinin‐positive cells; B), percent cardiomyocyte (α‐actinin‐positive cells/DAPI‐positive cells; C), and average green fluorescent area per cardiomyocyte (α‐actinin‐positive area/number of cardiomyocytes; D). Data are presented as mean ± SEM. *n* = 6, *P < 0.05, **P < 0.01, ***P < 0.001 vs. negative siRNA using Student’s *t*‐test.

At 72 h post‐DNMT1 siRNA transfection (12 nm), the total numbers of cells and cardiomyocytes were reduced by 24.4% and 20.8%, respectively (Fig. 1A,B), as compared to the negative control siRNA treatment. However, the percentage of cardiomyocytes in the culture remained unchanged after knockdown of DNMT1 (Fig. 1C). The average size of cardiomyocytes, as measured by α‐actinin‐positive area per cardiomyocyte, was increased by 56.9% after DNMT1 siRNA treatment (Fig. 1D).

### Down‐regulation of DNMT1 reduces beating rate and amplitude of field action potential in embryonic cardiomyocytes

**Fig. 2:**
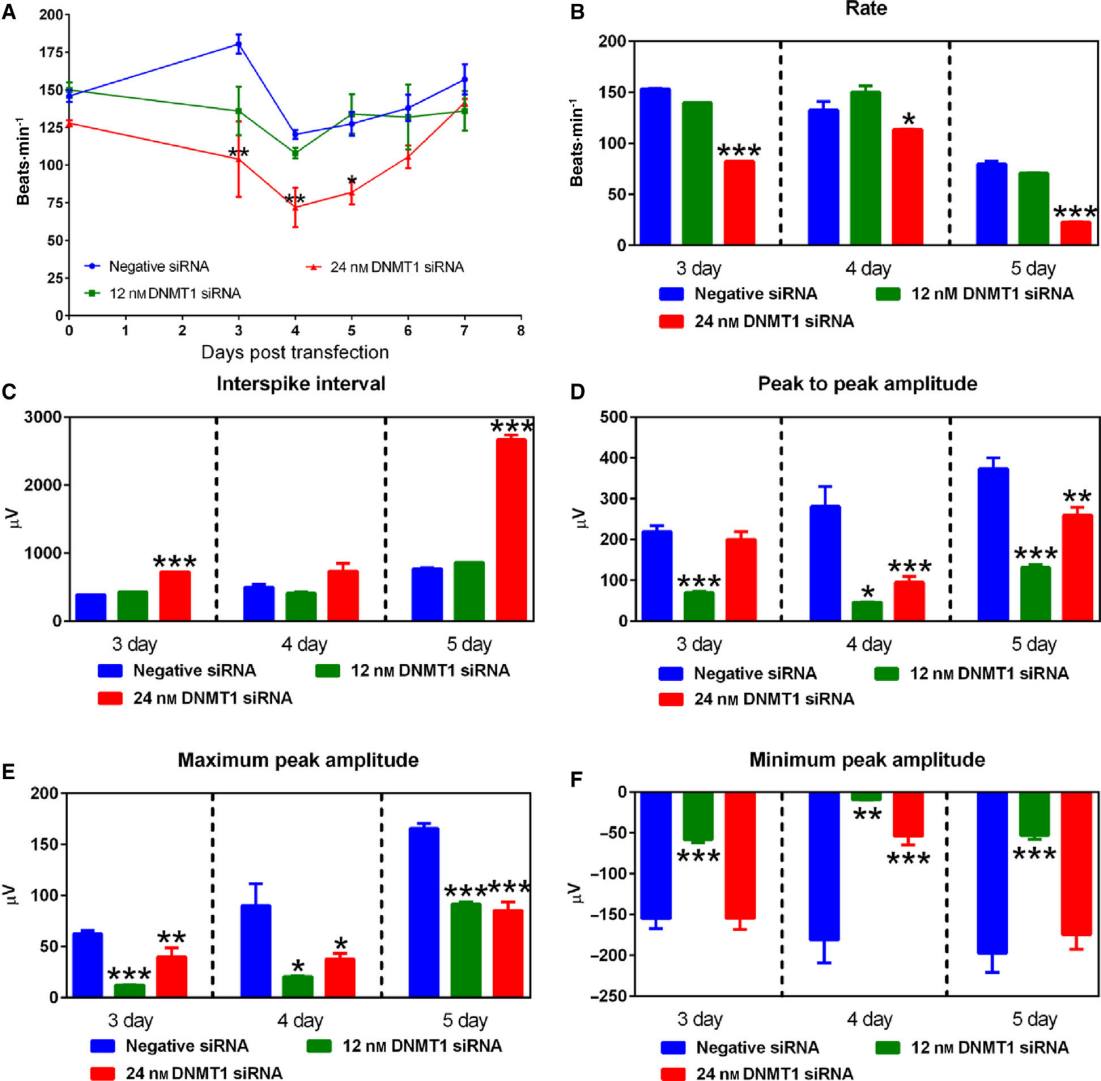
Knockdown of DNMT1 expression decreases the beating rate and amplitude of field action potential of embryonic cardiomyocytes. Counting cell contraction under a phase‐contrast microscope, we did not find significant changes in beating rate in the cells treated with 12 nm DNMT1 siRNA (A). But 24 nm DNMT1 siRNA significantly reduced the beating rate (A), which recovered over time as the knockdown effect subsided. The influence of 24 nm DNMT1 siRNA on beating rate was confirmed by multielectrode array (MEA) analysis (B and C). The MEA measurement also found reduced peak to peak amplitude (D), maximum peak amplitude (E), and minimum peak amplitude (F) following DNMT1 siRNA treatment at 12 and 24 nm. Data are presented as mean ± SEM. *n* = 3, *P < 0.05, **P < 0.01, ***P < 0.001 vs. negative siRNA using one‐way ANOVA followed by Neuman–Keuls *post hoc* test.

We next examined the contractile function and field action potential of cardiomyocytes following knockdown of DNMT1 expression. Counting cell contraction under a phase‐contrast microscope and a MEA2100 system, we found that DNMT1 siRNA at 24 nm reduced the beating rate of cardiomyocytes by 35.7–42.4% at 3–5 days post‐transfection (Fig. 2A). The beating rate gradually returned to normal by day 6 post‐siRNA transfection. The MEA2100 system also recorded reduced beating rate (Fig. 2B) and increased interspike interval (Fig. 2C) in these cells. DNMT1 siRNA treatment at 12 and 24 nm decreased the peak‐peak amplitude (Fig. 2D), maximum peak amplitude (Fig. 2E), and minimum peak amplitude (Fig. 2F) of the electrocardiogram.

### Differential gene expression in embryonic cardiomyocytes after knockdown of DNMT1 expression

**Fig. 3:**
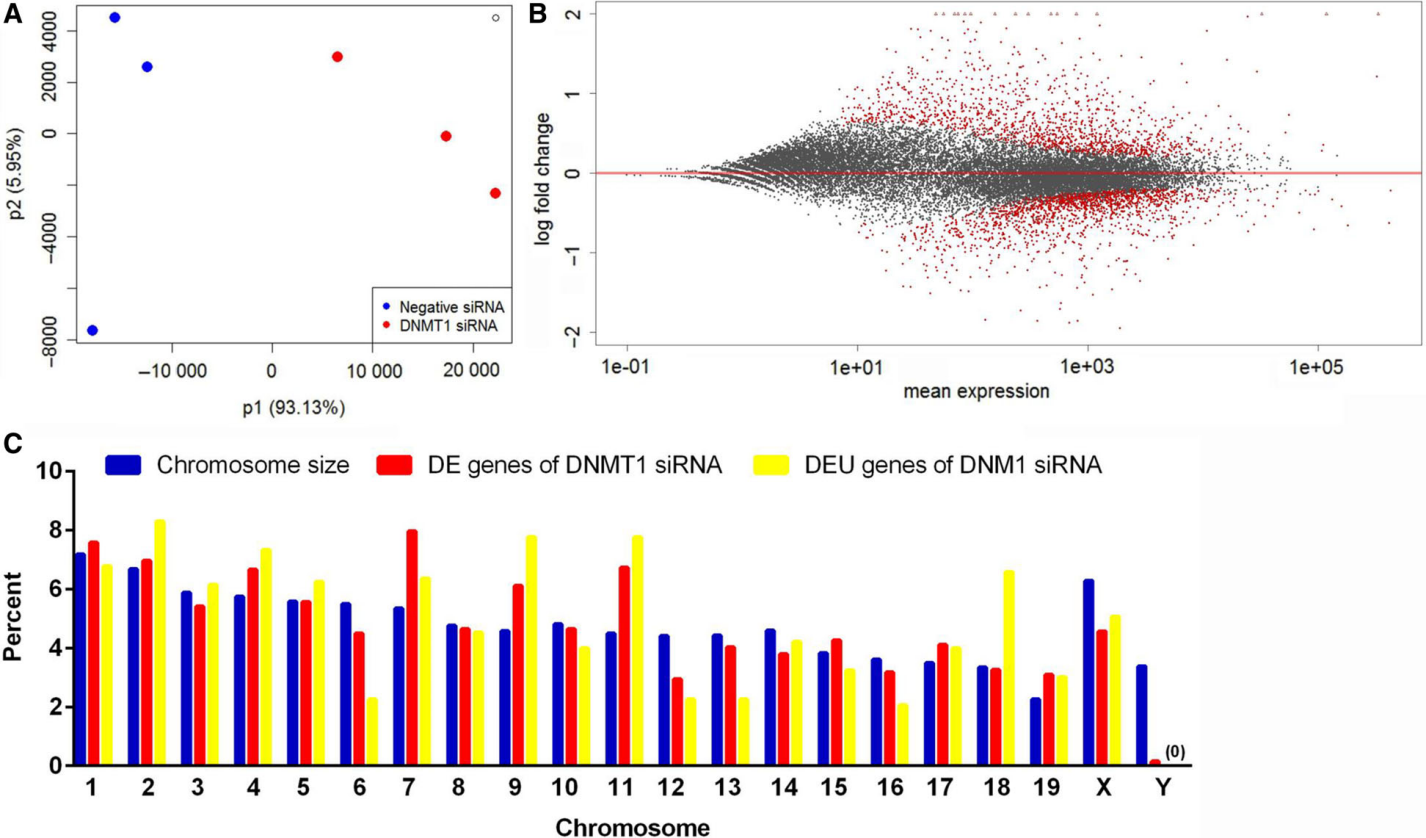
RNA‐Seq results. Principal component analysis (PCA) shows that the samples were well‐separated by treatment (A). The percent variability attributed to the first two principal components is displayed on the *X* and *Y* axes. The MA plot shows the expression changes of genes at 72 h following the knockdown of DNMT1 (12 nM; B). *X*‐axis is the mean raw counts of gene expression. *Y*‐axis is log2 fold change. Black dots represent nonsignificant genes, and red dots represent significant differentially expressed (DE) genes (false discovery rate [FDR] < 0.05 and absolute fold change >1.5). The percent DE and differentially exon usage (DEU) genes in each mouse chromosome are compared to the genome size (C). DE and DEU genes were distributed quite evenly across the homologous chromosomes.

To be consistent with our previous study of using DNMT siRNAs at 12 nm [10], we performed RNA‐Seq to examine the transcriptome of the embryonic cardiomyocytes at 72 h after transfection with 12 nm DNMT1 or negative siRNA. Our earlier flow cytometry data showed that the 95% of the magnetically sorted cells were cardiomyocytes [10]. The mapping efficiency of sequencing reads was above 80%, and principal component analysis showed that the samples were well clustered by treatments (Fig. 3A). DE analysis by deseq2 and edger software revealed that 801 genes were up‐regulated, and 494 genes were down‐regulated after knockdown of DNMT1 expression for 72 h (Fig. 3B, Table 1 and Table S1). These were a combined list of DE genes identified by deseq2 and edger. Expression changes of ten genes were confirmed by qPCR (Table 2).

**Table 1:** Differentially expressed (DE) genes or exons following knockdown of DNMT1 expression for 72 h in embryonic cardiomyocytes.

Method	# of up‐regulated genes/exons^a^	# of down‐regulated genes/exons^a^
DEseq2	619	432
EdgeR	776	481
Unique DE genes^b^	801	494
DEXseq	583 exons (488 genes)	346 exons (308 genes)

^a^ Genes or exons with false discovery rate (FDR) less than 0.05 and absolute fold change > 1.5 were considered as significant.

^b^ Combining the DEseq2 and EdgeR results, there were 801 unique genes that were up‐regulated and 494 unique genes that were down‐regulated.

**Table 2:** qPCR validation of gene expression data obtained by RNA‐Seq.

Symbol	Fold change (RNA‐Seq, EdgeR)^a^	Fold change (qPCR)
*Dnmt1*	−4.06*	−4.23*
*Dnmt3a*	1.18	1.26
*Dnmt3b*	1.17	1.20
*Myh6*	2.67*	2.67*
*Tnnc1*	2.51*	2.79*
*Tnni3*	2.93*	3.86*
*Tnnt2*	1.54*	1.59*
*Nppa*	3.16*	4.22*
*Nppb*	6.20*	9.52*
*Nkx2.5*	1.75*	1.97*

^a^ Genes with false discovery rate (FDR) < 0.05 and absolute fold change > 1.5 were considered as significant.

* *P* < 0.05 vs. negative siRNA treatment.

### DEU genes in embryonic cardiomyocytes after DNMT1 knockdown

**Fig. 4:**
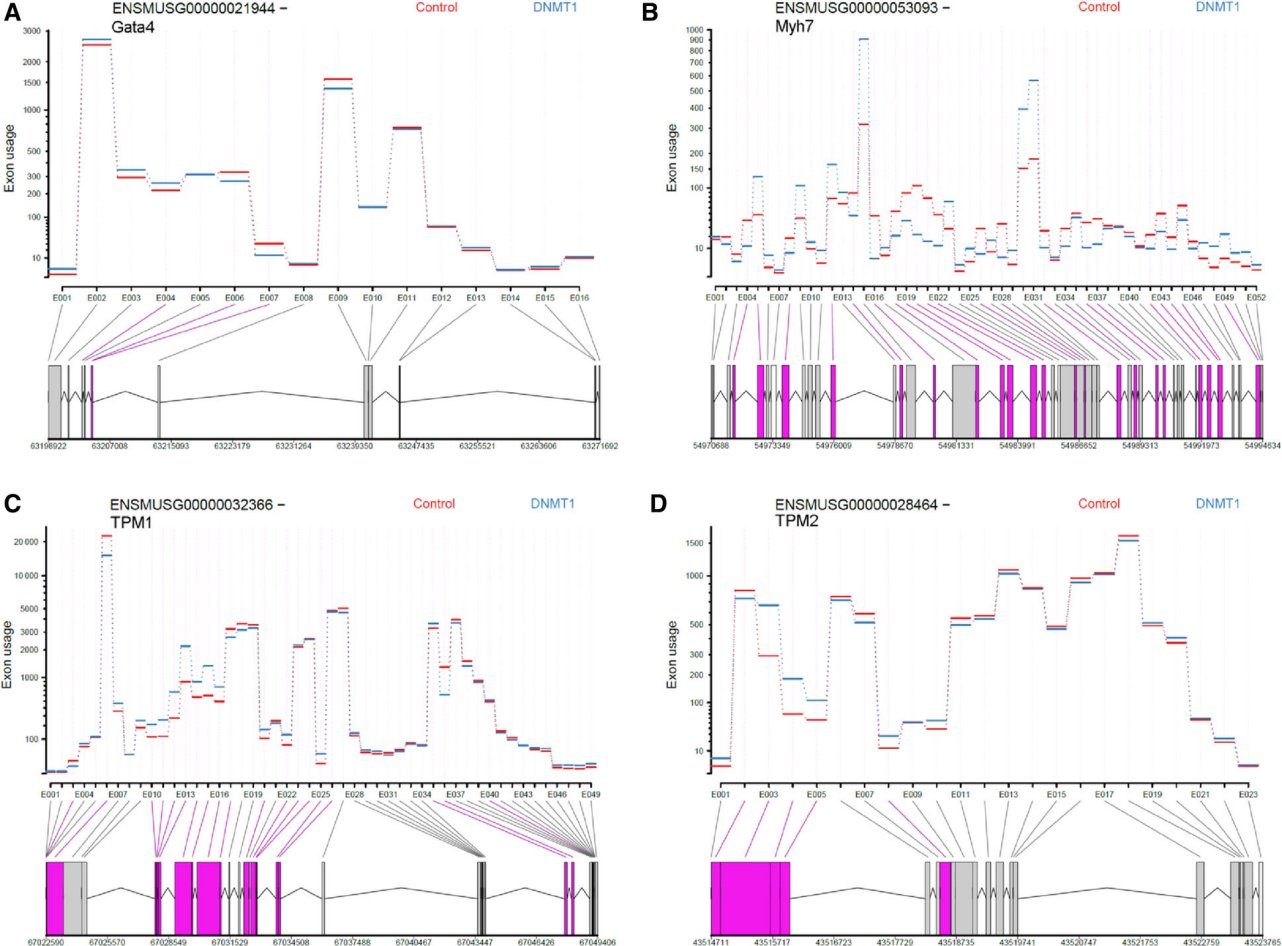
Differential exon usage (DEU) in cardiac genes. DEXseq analysis on RNA‐Seq data revealed that knockdown of DNMT1 expression for 72 h significantly altered exon usage of *Gata4* (A), *Myh7* (B), *Tpm1* (C), and *Tpm2* (D) in embryonic cardiomyocytes. The *x*‐axis shows the exons within a gene, and the *y*‐axis represents exon usage (normalized read counts of exons). Exons highlighted in purple are significant DEU exons with false discovery rate (FDR) <0.05 and absolute fold change >1.5.

It is known that DNA methylation in intragenic regions contributes to alternative splicing [16]. Thus, we studied the effects of reduced DNMT1 expression on alternative splicing, as indicated by different levels of exon usage. Analysis by DEXseq identified 929 exons that had differential usage levels after knocking down of DNMT1 (DEU exons), including 583 up‐regulated exons and 346 down‐regulated exons, following knockdown of DNMT1 expression for 72 h (Table 1 and Table S2). These exons belong to 796 unique genes. Fig 4 shows the representative cardiac genes with DEU exons, including *Gata4*, *Myh7*, *Tpm1*, and *Tpm2*. However, expression of these 4 genes was not changed at the gene level.

### Overlap of DE and DEU genes

We next compared the genes affected by altered splicing and the genes that were differentially expressed following knockdown of DNMT1 expression. We found that 59 DEU genes overlapped with DE genes, indicating that these genes were affected at both the transcription and splicing levels.

The distribution of DE genes and DEU genes across chromosome is shown in Fig 3C. DE genes were distributed quite evenly across the homologous chromosomes. But, the Y chromosome had less DE and DEU genes in proportion to its size. Interestingly, high percentages of DEU genes were found in chromosomes 2, 4, 9, 11, and 18.

### Pathway analysis of DE genes

Diseases and Bio Functions analysis of DE genes by IPA revealed that pathways related to cell death and survival, cell morphology, cell assembly and organization, and molecular transport were affected following DNMT1 siRNA treatment (Table 3). The cell death and survival pathways were predicted to be inactivated, and the assembly of intercellular junctions and organization of cytoskeleton pathways were predicted to be activated. In addition, several cardiac function and disease pathways were significantly enriched (Table 4). Interestingly, the pathways related to cardiac function were predicted to be activated, whereas the pathways related to cardiac diseases were mostly inactivated.

**Table 3:** Altered cellular morphology and function pathways in embryonic cardiomyocytes following DNMT1 siRNA treatment.

	*P*‐value	Activation *Z*‐score^a^	# of molecules^b^
Cell death and survival			
Cell death of heart	1.75E‐07	−1.918	36
Cell death	1.41E‐16	−1.483	372
Proliferation of cells	2.22E‐20	−1.018	414
Necrosis	4.98E‐14	−0.763	295
Cell survival	4.82E‐08	−0.470	156
Apoptosis	1.46E‐14	−0.467	297
Cell morphology, cellular assembly, and organization			
Fibrogenesis	5.37E‐06	2.076	53
Assembly of intercellular junctions	8.12E‐06	1.733	37
Organization of cytoskeleton	1.44E‐08	1.386	160
Differentiation of cells	1.04E‐17	1.024	275
Cell movement	2.42E‐15	0.572	255
Morphology of cells	1.49E‐20	–	256
Size of cells	1.72E‐07	–	60
Abnormal morphology of plasma membrane	3.61E‐06	–	31
Morphology of sarcomere	1.25E‐06	–	9
Molecular transport			
Transport of ion	1.14E‐06	3.248	62
Transport of metal	3.26E‐06	2.765	51
Transport of molecule	4.35E‐09	2.749	182
Transport of cation	8.62E‐06	2.643	49
Ion homeostasis of cells	5.69E‐06	1.658	70
Release of Ca2+	9.96E‐06	1.361	30
Quantity of Ca2+	8.24E‐07	1.231	54

^a^ Positive number means activation, and negative number means deactivation. – = the pathway was neither activated nor deactivated based on the patterns of gene expression. *Z*‐scores greater than 2 or smaller than −2 can be considered significant.

^b^ Genes with false discovery rate (FDR) less than 0.05 and absolute fold change > 1.5 were considered as significant.

**Table 4:** Altered cardiac function and disease pathways in embryonic cardiomyocytes following DNMT1 siRNA treatment. At the molecular level, IPA analysis revealed that knockdown of DNMT1 expression altered many canonical pathways that are related to regulation of cardiac function (Table 5). Specifically, the calcium signaling, protein kinase A signaling, Wnt/Ca^2+^ pathway, catecholamine biosynthesis, endothelin‐1 signaling, cAMP‐mediated signaling, and cardiac β‐adrenergic signaling were predicted to be activated based on the gene expression patterns.

	*P*‐value	Activation *Z*‐score^a^	# of molecules^b^
Cardiac function			
Heart rate	2.18E‐20	3.254	65
Function of heart	9.60E‐11	3.135	43
Function of cardiovascular system	2.90E‐15	2.892	69
Cardiogenesis	1.11E‐17	2.445	92
Cardiac output	9.74E‐04	2.425	8
Contraction of cardiac muscle	6.25E‐18	2.217	34
Formation of myofibrils	1.61E‐15	1.710	25
Proliferation of cardiomyocytes	4.16E‐02	1.480	9
Organization of sarcomere	1.17E‐14	1.114	20
Morphology of cardiovascular system	4.23E‐19	–	108
Cardiac disease			
Left ventricular dysfunction	4.47E‐10	−3.254	26
Congestive heart failure	3.76E‐08	−2.759	22
Fibrosis of heart	1.89E‐10	−2.694	36
Hypertrophy of heart chamber	1.01E‐10	−2.105	29
Supraventricular arrhythmia	4.54E‐09	−2.000	27
Failure of heart	2.10E‐07	−1.940	39
Congenital heart disease	6.93E‐04	−1.903	26
Dysfunction of heart	3.44E‐06	−1.842	16
Necrosis of cardiac muscle	1.30E‐06	−1.800	33
Arrhythmia	3.23E‐12	−1.421	43
Mass of heart	4.98E‐10	−1.166	29
Familial heart disease	2.60E‐14	−1.159	42
Infarction	1.35E‐08	−0.748	54
Hypertrophy of heart cells	5.39E‐07	−0.082	27
Dilated cardiomyopathy	1.23E‐11	0.089	40
Diastolic dysfunction	1.78E‐06	–	10

^a^ Positive number means activation, and negative number means deactivation. – = the pathway was neither activated nor deactivated based on the patterns of gene expression. Z‐scores greater than 2 or smaller than −2 can be considered significant.

^b^ Genes with false discovery rate (FDR) < 0.05 and absolute fold change > 1.5 were considered as significant.

**Fig. 5:**
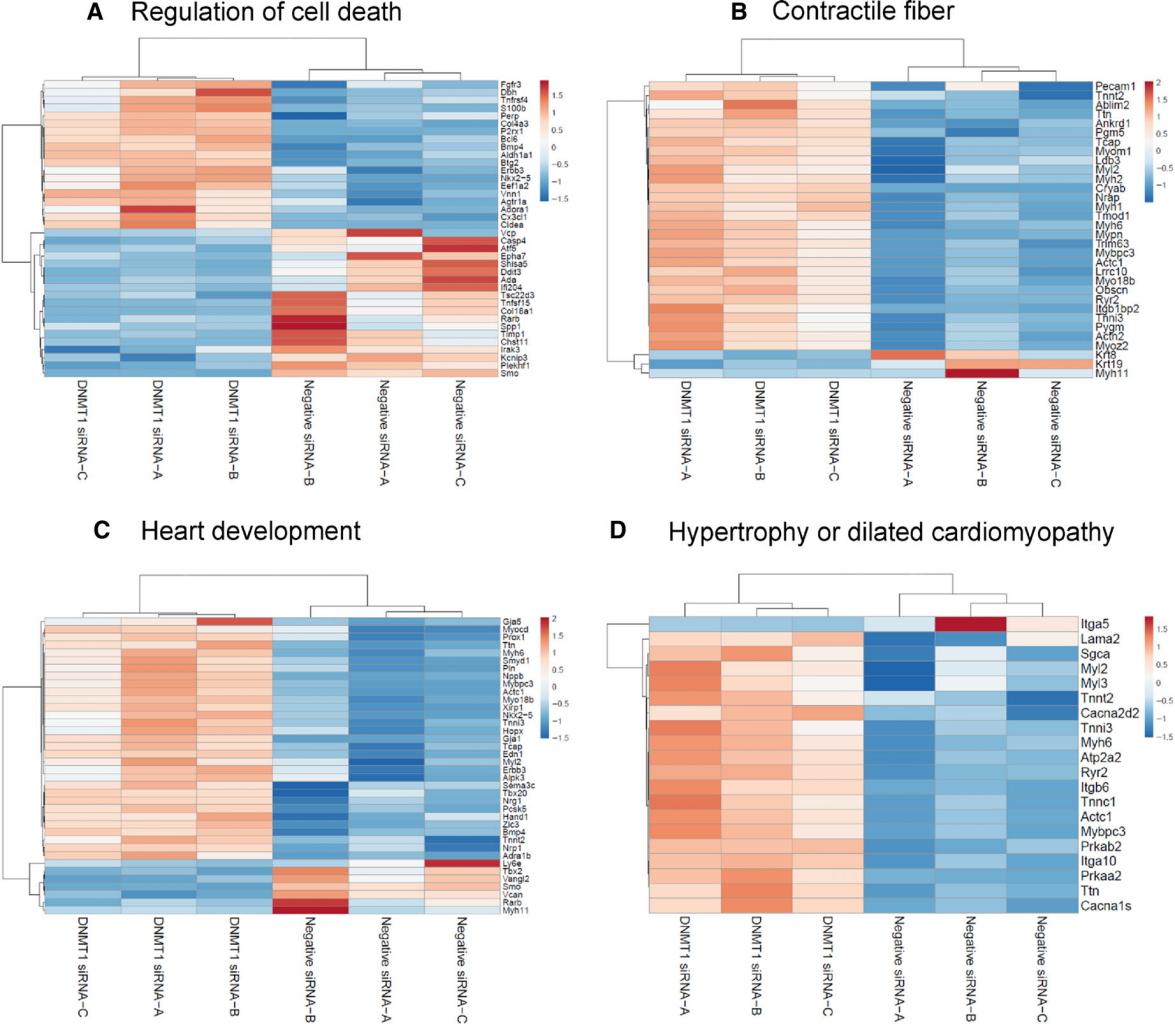
DNMT1 knockdown results in differential expression (DE) of genes in cell death and cardiac pathways. Heatmaps show the expression patterns of DE genes involved in the pathways of regulation of cell death (A), contractile fiber (B), heart development (C), and hypertrophy or dilated cardiomyopathy (D). Gene expression data (normalized raw counts) were centered and scaled by ClustVis before generating the heatmaps (orange, up‐regulated; blue, down‐regulated).

DAVID analysis on the DE genes also identified significantly altered pathways, with the heatmaps of gene expression patterns of the altered pathways shown in Fig. 5. Following DNMT1 siRNA treatment, the genes were most up‐regulated in the pathways related to formation of contractile fiber, heart development, and hypertrophy or dilated cardiomyopathy.

### Pathway analysis of DEU genes

DAVID analysis on the DEU genes demonstrated that they are involved in the pathways related to cell cycle, apoptosis, calmodulin binding, sarcomere, heart development, hypertrophic cardiomyopathy, etc. (Table 6).

**Table 5:** Significantly enriched cardiac and cell cycle pathways of DEU genes following DNMT1 siRNA treatment.

Pathways (category)	Ratio (%)^a^	*P*‐value	Genes
GO:0007049~cell cycle (GOTERM_BP_FAT)	6.20	3.92E‐04	LOC630896, E2F3, GM1859, GM9385, MLH1, CDC16, GM8416, WTAP, GM4870, GM8096, GM5847, GM13337, CDC45, GM5593, GM7743, ASPM, ANAPC1, CDK1, LOC675010, DDIT3, GM7380, PRDM9, GM7308, USP22, PDCD6IP, XRN1, NEK6, GM7669, ING4, CCDC99, ANAPC13, LOC100047393, POLA1, CHEK1, ITGB1, C79407, 4922501C03RIK, NUMA1, GM8341, RB1CC1, GM7390, CAMK2D, PKD2, BUB1, TFDP1, GM9252, PDS5B, LOC674691, GM5207, GM14292, GM6756, RPL24, RACGAP1, GM7901, EHMT2, ATM, NAE1, CCNB1, LOC100045322, GM9347, PHGDH, CALM3, GAS2L1, GM9210, CIT, SMC1A, CALM2, CALM1, MYH10
GO:0030017~sarcomere (GOTERM_CC_FAT)	1.51	0.002	MYO18B, ANK2, SLMAP, PDLIM5, SPNA2, MYH7, TTN, TPM2, CAPZB, TPM1
GO:0005516~calmodulin binding (GOTERM_MF_FAT)	1.66	0.008	LOC100048853, INVS, MYO1C, PHKA1, CAMK2D, ATPIF1, SPNA2, MYH7, MYH14, TTN, ASPM, MYH10
GO:0007507~heart development (GOTERM_BP_FAT)	2.57	0.010	PRKDC, TTN, FOXP4, ITGB1, TPM1, ATM, HDAC5, NOTCH1, MYO18B, D930049A15RIK, MYOCD, ZMIZ1, RB1CC1, GATA4, PKD2, SLC22A5, RBPJ, MYH10
s‐adenosyl‐l‐methionine (SP_PIR_KEYWORDS)	1.36	0.012	PRMT1, RNMT, PRDM9, EZH2, DNMT1, SETD7, CDKAL1, EHMT2, NSUN2
GO:0006915~apoptosis (GOTERM_BP_FAT)	4.08	0.026	LOC100047393, PRKDC, NFKB1, SCRIB, APLP1, RTN3, ANK2, SLK, TIA1, GM7390, TRAF7, ACIN1, CUL1, TFDP1, FAM82A2, AIMP2, LOC674691, TBRG4, MALT1, ATM, NAE1, LOC100046166, BBC3, TIAL1, LCK, TNFAIP8, SH3KBP1, MFSD10, PDCD6IP, EIF2AK2, NEK6
mmu05410:Hypertrophic cardiomyopathy (KEGG_PATHWAY)	1.21	0.038	ITGB8, ITGA10, ITGA3, MYH7, TTN, TPM2, ITGB1, TPM1

^a^ Percent of genes in the pathway that had differentially expressed exons (DEU). Exons with false discovery rate (FDR) < 0.05 and absolute fold change > 1.5 were considered as significant.

### Upstream transcription factors of DE genes

**Fig. 6:**
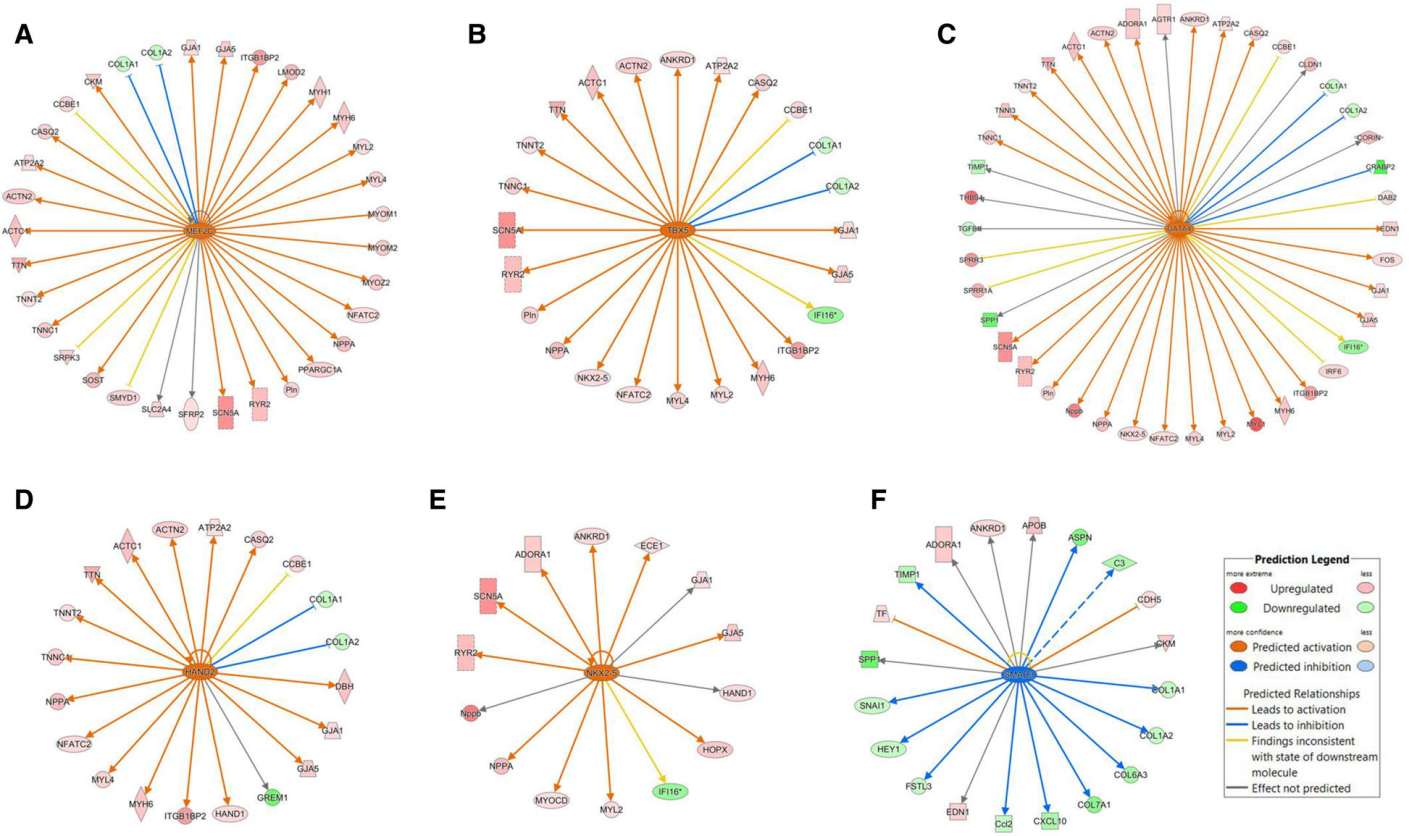
Cardiac transcription factors predicted to be activated or inhibited following knockdown of DNMT1 expression. Ingenuity pathway analysis (IPA) predicted that knockdown of DNMT1 expression activated Myocyte Enhancer Factor 2C (MEF2C; A), T‐Box Protein 5 (TBX5; B), GATA‐Binding Protein 4 (GATA4; C), Heart and Neural Crest Derivatives Expressed 2 (HAND2; D), and NK2 Homeobox 5 (NKX2.5; E) in primary cardiomyocytes. In contrast, transcription factor SMAD Family Member 3 (SMAD3) was predicted to be inhibited (F). Red or green represents a gene that was up‐ or down‐regulated, respectively, as detected by RNA‐Seq (Genes with false discovery rate [FDR] less than 0.05 and absolute fold change > 1.5 were considered as significant). Orange or blue means that the interaction between transcription factor and gene expression was predicted to be activated or inhibited, respectively. Yellow lines show the inconsistencies between our gene expression data and IPA predictions.

One mechanism for DNA methylation to inactivate gene expression is to suppress the binding of transcription factors to gene promoters. [14]. By analyzing the DE genes with the IPA software, we identified the transcription factors that may have increased or suppressed activities after knockdown of DNMT1 expression, even though they may not necessarily be altered at the transcription level (Table 7). The analysis predicted activation of transcription factors known to be related to cardiac gene regulation, including MEF2C, TBX5, GATA4, HAND2, MYOCD, MEF2A, NKX2.5, MYOD1, NKX2.3, and GATA5. Fig 6 shows the expression changes of target genes potentially mediated by MEF2C, TBX5, GATA4, HAND2, NKX2.5, and SMAD3. These transcription factors are linked to development of congenital heart disease [47, 48, 49].

**Table 6:** Predicted activation or suppression of transcription factors following knockdown of DNMT1 expression for 72 h in embryonic cardiomyocytes.

Transcription factor	Fold change^a^	Predicted activation state	Activation * z*‐score^b^	*P*‐value of overlap	# of target genes
TRIM24	1.646	Activated	5.807	1.23E‐21	35
MEF2C	–	Activated	4.602	2.20E‐20	33
TBX5	–	Activated	4.132	2.34E‐15	24
GATA4	–	Activated	4.001	8.70E‐20	42
HAND2	–	Activated	3.880	3.71E‐14	20
SRF	–	Activated	3.219	2.90E‐12	49
MYOCD	1.516	Activated	2.889	2.41E‐17	28
MEF2A	–	Activated	2.760	7.99E‐13	15
NKX2‐5	1.754	Activated	2.735	1.05E‐10	15
SPDEF	–	Activated	2.449	4.77E‐02	7
YAP1	–	Activated	2.425	5.66E‐03	9
ARNT2	–	Activated	2.400	1.20E‐03	21
SIM1	–	Activated	2.400	1.71E‐03	21
PPARGC1A	2.146	Activated	2.344	1.97E‐05	26
CREBBP	–	Activated	2.286	1.70E‐08	46
MYOD1	–	Activated	2.269	1.74E‐09	31
NKX2‐3	–	Activated	2.253	2.06E‐13	40
GLIS2	–	Activated	2.236	1.39E‐03	5
GATA5	–	Activated	2.219	9.61E‐06	6
RB1	–	Activated	2.178	2.78E‐03	31
IRF4	–	Activated	2.177	7.86E‐06	21
STAT5B	–	Activated	2.046	1.86E‐09	29
NFKB1	–	Activated	2.032	4.98E‐05	27
FOS	1.644	Inhibited	−2.076	6.30E‐05	47
SNAI1	−1.531	Inhibited	−2.171	7.12E‐03	11
TBX3	–	Inhibited	−2.219	4.58E‐05	6
Foxp1	–	Inhibited	−2.449	2.04E‐05	9
SMAD3	–	Inhibited	−2.494	4.74E‐04	23
STAT2	−1.681	Inhibited	−2.589	7.37E‐05	10
NRIP1	–	Inhibited	−2.621	2.70E‐03	9
KDM5A	–	Inhibited	−2.668	2.02E‐04	17
PML	–	Inhibited	−2.751	3.47E‐02	11
STAT1	–	Inhibited	−2.861	1.04E‐12	45
NFATC2	1.730	Inhibited	−2.982	8.76E‐06	21
ATF4	–	Inhibited	−3.272	4.21E‐08	24
HTT	–	Inhibited	−3.325	3.52E‐10	74
IRF5	–	Inhibited	−3.477	1.57E‐07	16
IRF1	–	Inhibited	−3.676	1.44E‐03	17
IRF3	–	Inhibited	−3.931	7.66E‐10	30
IRF7	−3.370	Inhibited	−4.738	1.64E‐14	36

^a^ Genes with false discovery rate (FDR) < 0.05 and absolute fold change > 1.5 were considered as significant.

^b^ Z‐scores greater than 2 or smaller than −2 can be considered significant.

### DNA methylation changes in embryonic cardiomyocytes after DNMT1 knockdown

Previously, we found that global cytosine methylation level is significantly reduced by DNMT1 siRNA at 72 h post‐siRNA transfection [10]. In this study, we specifically examined the DNA methylation changes in target genes following knockdown of DNMT1 using multiplex targeted bisulfite sequencing.

We analyzed promoter methylation of 15 genes that are related to cardiomyocyte morphology and proliferation. Two proximal promoter (< 3000 bp from transcription start site) regions of each gene were interrogated. Consistent with global DNA demethylation, decreased promoter methylation was identified in the target genes. Following knockdown of DNMT1 for 72 h, decreased methylation was observed in the promoter CpG sites of 13 target genes, including *Myh6*, *Myh7*, *Myh7b*, *Tnnc1*, *Tnni3*, *Tnnt2*, *Nppa*, *Nppb*, *mef2c*, *mef2d*, *Camta2*, *Cdkn1A*, and *Cdkn1C* (Table 8). Of these 13 differentially methylated genes, 6 (*Myh6*, *Tnnc1*, *Tnni3*, *Tnnt2*, *Nppa*, *Nppb*) had increased gene expression, 1 (*Cdkn1C*) had decreased gene expression, and 6 (*Myh7*, *Myh7b*, *mef2c*, *mef2d*, *Camta2*, *Cdkn1C*) had no change in gene expression in the DNMT1 knockdown cells when compared to the control cells. Therefore, methylation may be one of multiple functions contributing to expression regulation of these cardiac genes.

**Table 7:** Methylation changes in promoter CpGs following knockdown of DNMT1 expression for 72 h.

Gene^a^	CpG location in amplicon	FDR of methylation change	% methylation difference	Fold change in gene expression (RNA‐Seq)^b^	FDR of fold change^b^
*Myh6_1*	43 (#1)	0	−9.51	2.67	6.83E‐15
*Myh6_1*	71 (#2)	0	−5.15		
*Myh6_1*	132 (#3)	0	−5.02		
*Myh6_1*	362 (#4)	0	−17.45		
*Myh6_2*	68 (#1)	0	−20.39		
*Myh6_2*	170 (#2)	0	−23.06		
*Myh6_2*	202 (#3)	0	−20.38		
*Myh7_1*	66 (#1)	0	−16.57	1.16	0.761
*Myh7_1*	102 (#2)	0	−12.29		
*Myh7_1*	118 (#3)	0	−13.64		
*Myh7_1*	127 (#4)	0	−13.86		
*Myh7_1*	202 (#5)	0	−9.80		
*Myh7_1*	234 (#6)	0	−11.66		
*Myh7_1*	285 (#7)	0	−17.79		
*Myh7_1*	290 (#8)	0	−16.61		
*Myh7_1*	308 (#9)	0	−17.47		
*Myh7_2*	225 (#4)	0	−5.30		
*Myh7b_1*	145 (#2)	0	−21.23	1.16	0.825
*Myh7b_1*	161 (#3)	0	−14.75		
*Myh7b_2*	122 (#1)	0	−13.09		
*Myh7b_2*	167 (#2)	0	−15.01		
*Myh7b_2*	248 (#3)	0	−9.11		
*Myh7b_2*	352 (#4)	0	−14.69		
*Tnnc1_1*	46 (#1)	0	−14.12	2.51	4.00E‐15
*Tnnc1_1*	114 (#2)	0	−15.63		
*Tnnc1_1*	198 (#3)	0	−22.16		
*Tnnc1_1*	240 (#4)	0	−7.28		
*Tnnc1_1*	243 (#5)	0	−6.97		
*Tnnc1_1*	255 (#6)	0	−7.72		
*Tnnc1_2*	107 (#4)	0	−17.23		
*Tnnc1_2*	196 (#5)	0	−5.21		
*Tnnc1_2*	272 (#6)	0	−10.44		
*Tnnc1_2*	299 (#7)	0	−11.86		
*Tnnc1_2*	340 (#8)	0	−12.16		
*Tnnc1_2*	365 (#9)	0	−13.99		
*Tnni3_1*	66 (#1)	0	−15.13	2.93	2.60E‐10
*Tnni3_1*	103 (#2)	0	−14.92		
*Tnni3_1*	171 (#3)	0	−7.67		
*Tnni3_1*	194 (#4)	0	−12.04		
*Tnni3_1*	244 (#5)	0	−11.00		
*Tnni3_1*	355 (#6)	0	−13.10		
*Tnni3_1*	409 (#7)	2.70E‐08	−13.92		
*Tnni3_2*	49 (#1)	7.68E‐14	−25.14		
*Tnni3_2*	62 (#2)	4.36E‐11	−18.97		
*Tnni3_2*	200 (#4)	0	−11.31		
*Tnni3_2*	321 (#5)	0	−12.93		
*Tnni3_2*	354 (#6)	0	−11.39		
*Tnni3_2*	398 (#7)	0	−18.29		
*Tnnt2_1*	94 (#1)	0	−12.21	1.54	1.36E‐04
*Tnnt2_1*	118 (#2)	0	−14.66		
*Tnnt2_1*	195 (#3)	0	−24.45		
*Tnnt2_1*	201 (#4)	0	−16.42		
*Tnnt2_1*	350 (#5)	0	−12.36		
*Tnnt2_1*	420 (#6)	3.28E‐15	−5.45		
*Tnnt2_2*	58 (#1)	0	−13.55		
*Tnnt2_2*	66 (#2)	0	−13.72		
*Tnnt2_2*	209 (#3)	0	−21.33		
*Tnnt2_2*	224 (#4)	0	−11.15		
*Tnnt2_2*	233 (#5)	0	−16.13		
*Tnnt2_2*	240 (#6)	0	−11.96		
*Tnnt2_2*	263 (#7)	0	−15.42		
*Tnnt2_2*	288 (#8)	0	−25.92		
*Nppa_1*	125 (#3)	0	−8.03	3.16	9.12E‐19
*Nppa_1*	133 (#4)	0	−5.36		
*Nppa_1*	233 (#5)	0	−11.81		
*Nppa_1*	296 (#6)	0	−10.91		
*Nppa_1*	303 (#7)	0	−9.71		
*Nppa_1*	310 (#8)	0	−16.30		
*Nppa_1*	352 (#9)	0	−14.00		
*Nppa_1*	405 (#10)	0	−10.50		
*Nppb_2*	51 (#1)	0	−19.68	6.20	1.82E‐44
*Nppb_2*	238 (#2)	0	−12.03		
*Nppb_2*	312 (#3)	0	−19.09		
*Nppb_2*	328 (#4)	0	−12.86		
*Nppb_2*	341 (#5)	0	−17.78		
*Nppb_2*	381 (#6)	1.82E‐16	−8.28		
*Mef2c_1*	64 (#1)	0	−15.92	1.01	0.973
*Mef2c_1*	115 (#2)	0	−17.19		
*Mef2c_1*	166 (#3)	0	−24.23		
*Mef2c_1*	268 (#4)	0	−26.42		
*Mef2c_1*	315 (#5)	0	−19.21		
*Mef2c_1*	348 (#6)	0	−19.07		
*Mef2d_1*	26 (#1)	9.15E‐05	−11.30	1.14	0.271
*Mef2d_2*	50 (#1)	4.58E‐15	−11.22		
*Mef2d_2*	82 (#2)	0	−11.16		
*Mef2d_2*	261 (#5)	0	−6.93		
*Camta2_2*	185 (#2)	0	−6.35	1.27	0.038
*Camta2_2*	196 (#3)	0	−8.96		
*Camta2_2*	247 (#4)	0	−7.84		
*Camta2_2*	299 (#5)	0	−6.04		
*Cdkn1A_1*	84 (#1)	0	−23.45	1.14	0.348
*Cdkn1A_1*	124 (#2)	0	−19.09		
*Cdkn1A_1*	222 (#3)	0	−18.23		
*Cdkn1A_1*	374 (#4)	1.07E‐10	−5.33		
*Cdkn1A_1*	393 (#5)	0	−20.13		
*Cdkn1C_1*	24 (#1)	0.008009	−5.04	−1.84	0.003
*Cdkn1C_1*	33 (#3)	5.07E‐06	−7.34		
*Cdkn1C_1*	40 (#4)	2.37E‐12	−9.71		
*Cdkn1C_1*	60 (#7)	4.95E‐07	−6.36		
*Cdkn1C_1*	66 (#8)	9.49E‐14	−8.01		
*Cdkn1C_1*	69 (#9)	3.10E‐10	−8.33		
*Cdkn1C_1*	96 (#10)	1.73E‐08	−6.80		
*Cdkn1C_1*	104 (#11)	1.96E‐12	−8.85		
*Cdkn1C_1*	106 (#12)	6.15E‐12	−8.30		
*Cdkn1C_1*	108 (#13)	1.00E‐06	−5.78		
*Cdkn1C_1*	113 (#15)	1.25E‐10	−7.12		
*Cdkn1C_1*	115 (#16)	1.09E‐11	−7.99		
*Cdkn1C_1*	123 (#18)	2.86E‐14	−9.28		
*Cdkn1C_1*	213 (#29)	0	−6.22		
*Cdkn1C_1*	258 (#34)	0	−5.05		
*Cdkn1C_1*	265 (#36)	0	−7.73		
*Cdkn1C_1*	269 (#37)	0	−6.74		
*Cdkn1C_1*	273 (#38)	0	−7.19		
*Cdkn1C_1*	287 (#40)	0	−9.94		
*Cdkn1C_1*	291 (#41)	0	−7.40		
*Cdkn1C_1*	313 (#45)	0	−5.61		
*Cdkn1C_1*	340 (#48)	3.20E‐11	−6.52		
*Cdkn1C_1*	391 (#53)	0.044921	5.71		

^a^ These target genes were selected because they are important genes related to cardiomyocyte morphology and proliferation.

^b^ Genes with false discovery rate (FDR) < 0.05 and absolute fold change > 1.5 were considered as significant.

To predict the effect of decreased promoter methylation on transcription factor binding, we used the promo software to identify the transcription factor binding sites in the promoter regions of the target genes that had altered methylation. We next investigated the activation status of these transcription factors by using the IPA upstream analysis results. The altered cardiac genes, namely *Myh6*, *Tnnc1*, *Tnni3*, *Tnnt2*, *Nppa*, *Nppb*, and *Camta2*, shared several common transcription factors in their promoters (Table 9). Of these transcription factors, SRF, NKX2.5, ARNT, MYOD, MEF2C, and STAT5B were predicted to be activated following DNMT1 siRNA treatment. These results suggested that decreased methylation levels in these promoter sequences may facilitate transcription factor binding and trigger downstream gene expression.

**Table 8:** PROMO software analysis identified IPA‐predicted transcription factor binding sites in the proximal promoter of cardiac genes.

Gene	Transcription factor (TF) binding sites in proximal promoter (< 3000 bp upstream of transcription start site)	
	TFs predicated to be activated^a^	TFs predicated to be inhibited^a^
*Myh6*	SRF, NKX2‐5, ARNT, MYOD	c‐FOS, SMAD3, STAT1, IRF1, IRF3
*Tnnc1*	MEF2C, SRF, NKX2‐5, ARNT, MYOD	c‐FOS, IRF1, IRF3
*Tnni3*	SRF, NKX2‐5, ARNT, MYOD	c‐FOS, STAT1, IRF1, IRF3, IRF7
*Tnnt2*	SRF, NKX2‐5, ARNT, MYOD	c‐FOS, SMAD3, STAT1, IRF1, IRF3
*Nppa*	MEF2C, SRF, NKX2‐5, ARNT, MYOD	c‐FOS, SMAD3, STAT1, IRF1, IRF3, IRF7
*Nppb*	SRF, NKX2‐5, ARNT, MYOD, STAT5B	c‐FOS, SMAD3, STAT1, IRF1, IRF3
*Camta2*	MEF2C, SRF, NKX2‐5, ARNT, MYOD, STAT5B	c‐FOS, SMAD3, STAT1, IRF1, IRF3

^a^ Prediction is based on the Ingenuity pathway analysis (IPA) of the RNA sequencing data.

## Discussion

DNMTs are important enzymes to establish and maintain DNA methylation patterns, deletion of which may cause embryonic lethality [13, 50]. To date, the role of DNMTs in the heart remains largely unknown. Recently, DNMT1 is found to be up‐regulated in the atrium of rats with isoproterenol‐induced heart failure [51]. Myocardial tissue‐specific DNMT1 knockout in rats protects against pathological injury induced by Adriamycin [36]. These findings suggest that DNMT1 plays an important role in the development and progression of heart diseases and decreased DNMT1 expression may protect the heart function. However, the direct effect of DNMT1 expression on cardiomyocytes remains unclear. We now show that knockdown of DNMT1 causes significant changes in DNA methylation patterns, gene expression (including deactivation of the heart disease pathways), and function of embryonic cardiomyocytes.

One of our previous studies found that knockdown of DNMT3a alters gene expression and decreases beating frequency, contractile movement, amplitude of field action potential, and cytosolic calcium signaling of embryonic cardiomyocytes [10]. We also found that down‐regulation of DNMT1 by siRNA markedly increases cytotoxicity and apoptosis, resulting in decreased cell viability [10]. Consistent with our early findings, the data from the present study reveal that DNMT1 siRNA treatment for 72 h significantly reduces the number of cardiomyocytes as compared to the control siRNA. Other reports also show that inhibition of DNMT1 decreases cell numbers and promotes apoptosis in other cell types [52, 53, 54]. The RNA‐Seq results demonstrate that the cell survival and proliferation pathways are deactivated following DNMT1 siRNA treatment, which may explain the reduced cell numbers (Table 3). Interestingly, the cell apoptosis and necrosis pathways are also deactivated (Table 3), which may be a compensatory change to inhibit cell death in response to the reduction in cell number. Furthermore, DNMT1 siRNA treatment increases the size of cardiomyocytes, which may be due to the changes in gene expression involved in fibrogenesis, size of cells, and morphology of sarcomere (Table 3). Functionally, DNMT1 siRNA significantly reduces the beating rate of cardiomyocytes and decreases the amplitude of field action potential. However, the genes involved in the contractile fiber are mostly up‐regulated by DNMT1 siRNA (Fig. 5B), which may be a cellular compensatory effort to synthesize contractile fibers in response to the decreased contractility.

The above results show that DNMT1 plays an essential role in regulating the cell cycle, size, and function of cardiomyocytes, and therefore, deregulation of DNMT1 may contribute to the onset of cardiovascular diseases (CVD). It is known that many CVD risk factors, such as stress, pollution, smoking, *in utero* undernutrition, and circadian rhythm, have been associated with modification of DNA methylation marks [55]. Patients with atherogenesis, coronary artery disease, dilated cardiomyopathy, and heart failure have abnormal patterns of DNA methylation [28, 29, 30, 31, 32, 33, 34]. Therefore, proper DNMT1 activity and DNA methylation patterns are key to the health of cardiomyocytes.

We previously found that down‐regulation of DNMTs, including DNMT1, in embryonic ventricles by *in utero* caffeine exposure correlates with altered DNA methylation patterns, gene expression profiles, and cardiac function in adult mice [37]. *In utero* caffeine exposure up‐regulates the expression of cardiac genes, including *Myh6*, *Tnni3*, *Nppa*, and *Nppb*, which are also increased following the knockdown of DNMT1 in this study. Therefore, DNMT1 may be involved in mediating the *in utero* caffeine effects on gene expression in the embryonic heart.

Our RNA‐Seq results predicted the activation of pathways related to cardiac function (Table 4). These predictions are based on the expressional changes of genes involved in the molecular pathways including but not limited to the activation of the cAMP‐mediated signaling, calcium signaling, endothelin‐1 signaling, and cardiac β‐adrenergic signaling (Table 5). The gene expression patterns seem to be contradictive to the observed decreased beating rate and amplitude of field action potential, and therefore, these changes may be the compensatory mechanisms in the cardiomyocytes to overcome the reduced function following DNMT1 siRNA treatment. Our RNA‐Seq results also predicted deactivation of the cardiac disease pathways, which may explain the protective effects against Adriamycin‐induced pathological injury in the heart of myocardial tissue‐specific DNMT1 knockout rats [36].

**Table 9:** Altered canonical cardiac pathways in embryonic cardiomyocytes following DNMT1 siRNA treatment.

Ingenuity canonical pathways	*P*‐value	Ratio (%)^a^	Activation *Z*‐score^b^
ILK signaling	1.12E‐04	12.4	2.558
Wnt/Ca+ pathway	2.40E‐03	16.1	2.333
cAMP‐mediated signaling	4.07E‐02	8.2	2.183
Actin cytoskeleton signaling	2.69E‐05	12.5	1.886
Calcium signaling	5.50E‐08	16.3	1.604
Protein kinase A signaling	1.58E‐04	9.8	1.461
PAK signaling	7.41E‐04	14.6	1.387
Regulation of actin‐based motility by Rho	2.09E‐02	11.0	1.265
Endothelin‐1 signaling	1.95E‐02	9.3	1.155
Cardiomyocyte differentiation via BMP receptors	3.16E‐03	25.0	1.000
Cardiac β‐adrenergic signaling	4.79E‐02	9.0	1.000
Nitric oxide signaling in the cardiovascular system	6.17E‐03	12.0	−0.302
Ephrin receptor signaling	1.05E‐02	9.8	−1.414
Interferon signaling	2.40E‐03	19.4	−2.646
Tight junction signaling	7.08E‐03	10.2	–
Factors promoting cardiogenesis in vertebrates	8.91E‐03	12.0	–
G‐protein coupled receptor signaling	8.91E‐03	9.0	–
Ephrin A signaling	1.23E‐02	14.6	–
Catecholamine biosynthesis	1.55E‐02	50.0	–
eNOS signaling	1.74E‐02	9.9	–

^a^ Percent of genes in the pathway that were changed in expression. Genes with false discovery rate (FDR) < 0.05 and absolute fold change > 1.5 were considered as significant.

^b^ Positive number means activation, and negative number means deactivation. – = the pathway was neither activated nor deactivated based on the patterns of gene expression. *Z*‐scores greater than 2 or smaller than −2 can be considered significant.

Our previous finding has shown that knockdown of DNMT1 expression decreases global cytosine methylation level in the genomic DNA [10]. Consistently, through next‐generation sequencing, we find in this study that decreased DNMT1 expression results in reduced promoter methylation levels in 13 out of 15 target genes, which are related to cardiomyocyte morphology and proliferation. Changes in promoter methylation may have contributed to increased gene expression of 6 target genes. These data suggested that methylation may be one of multiple functions contributing to expression regulation of these cardiac genes. With the RNA‐Seq data, we predicted the impact of promoter demethylation on transcription factor activities (Table 7), the changes of which may lead to cascade effects on gene expression. Additionally, DNMT1 siRNA affects gene splicing in many genes, which may be due to the alterations in gene body methylation levels [56]. Collectively, these data demonstrate the direct effects of DNMT1 suppression on the maintenance of methylation patterns and proper gene expression and splicing.

Overall, we now demonstrate that knockdown of DNMT1 changes promoter methylation patterns, alters cardiac gene expression and splicing, and inhibits cell viability, beating frequency, and amplitude of field action potential in embryonic cardiomyocytes. Collectively, these data demonstrate that DNMT1 plays an important role in regulating cardiomyocyte DNA methylation, gene expression, and function.

## Conflicts of interest

The authors declare no conflict of interest.

## Author contributions

XF, CCW, and SAR conceived and designed the project, XF and RP acquired the data, XF analyzed the data, XF, LZ, and JW interpreted the data, and XF, LZ, JW, and CCW wrote the paper.

## Supporting information


**Table S1**. DE genes supplementary table.

**Table S2**. DUE exons supplementary table.

## Data Availability

All RNA‐Seq data were uploaded to the Gene Expression Omnibus (GEO), and the accession number is GSE81446. All bisulfite‐Seq data were uploaded to GEO, and the accession number is GSE81464.
